# Comparison of landing kinematics and kinetics between experienced and novice volleyball players during block and spike jumps

**DOI:** 10.1186/s13102-022-00496-0

**Published:** 2022-06-11

**Authors:** Sébastien Garcia, N. Delattre, E. Berton, G. Divrechy, G. Rao

**Affiliations:** 1 Movement Sciences Department, Decathlon SportsLab, 59000 Lille, France; 2 CNRS, Insitute of Movement Sciences, Aix-Marseille University, 13007 Marseille, France

**Keywords:** Sport, Biomechanics, Landing, Experience, ACL injury

## Abstract

**Background:**

The practice of volleyball requires many jumps. During landing, anterior cruciate ligament injuries may occur with high-risk lower limb kinematics and kinetics. Differences in landing strategies between experienced and novice volleyball players have not been fully explored. The purpose of the study was to compare lower limb kinematics and kinetics in experienced and novice volleyball players when performing volleyball specific jumps.

**Methods:**

A total of 30 healthy males, 15 experienced and 15 novice volleyball players, participated in the study. Participants performed block and spike jumps at a controlled jump height. Hip, knee and ankle joints angles at initial ground contact and ranges of motion in the sagittal plane, knee joint angles and moments in the frontal plane, vertical ground reaction force peak and loading rate were analyzed to investigate the expertise effect.

**Results:**

Experienced volleyball players landed with larger ankle dorsiflexion range of motion compared to novices. For the spike jump, experienced players landed with larger ankle plantarflexion angles at initial contact and larger ankle dorsiflexion ranges of motion, and for the block jump, they landed with larger knee flexion ranges of motion. Experienced players jumped significantly higher than novices. No difference was found in vertical ground reaction force peaks and loading rates.

**Conclusions:**

Although the experienced group jumped higher than the novice group, no difference was found in ground reaction force parameters. These findings highlight that the experience of volleyball players acquired during regular trainings and competitions may play an important role in landing kinematics and kinetics to reduce the injury risk.

**Supplementary Information:**

The online version contains supplementary material available at 10.1186/s13102-022-00496-0.

## Background

Volleyball is a complex and demanding sport in terms of technique, tactics, and athleticism. The landing occurs mainly after block or spike jumps corresponding to the defensive and offensive maneuvers, respectively. Volleyball players performed a large volume of jumps with an average of 83 jumps per training session and 71 jumps per match [1]. Nonetheless, it has been reported that most of volleyball injuries occurred during block and spike jumps [2]. A recent study showed that 75% of anterior cruciate ligament (ACL) injuries that occur during volleyball practice are associated with the jump-landing phase and 86.5% are observed in non-contact situations [3]. ACL injuries are among the most severe and common knee injuries in volleyball, and often requires surgery [4].

A prospective study has shown that the risks of acute volleyball injury were associated with the volleyball experience [5]. In this study, 649 volleyball players involving first division and local division were observed during the 2005–2006 season. The follow-up revealed that the elite players reported 1.89 injuries per 1000 h of training or game per player, while local division’s players reported 2.8 injuries per 1000 h of training or game per player. Based on these findings, the players’ experience and skill in volleyball technique possibly reduces the risk of injury. Various factors may explain these results such as better training, the experience of coaches, the accessibility to medical care or complete rehabilitation [5]. Previous studies revealed that the landing strategy of athletes may be improved by a proper training intervention to decrease the occurrence of volleyball injuries [6–8]. This suggested that volleyball players' experience acquired during regular trainings and competitions may influence the landing strategy.

There are several biomechanical factors that can contribute to an increased risk of ACL injuries. It has been shown that landing with decreased hip and knee joints flexion, increased knee joint abduction angle at initial contact, increased peak vertical ground reaction force (GRF) and increased knee joint abduction moments may be linked to an increased risk of ACL injuries [9, 10]. A video-based analysis identified that athletes landed with the rearfoot or in a flatfooted position at the time of ACL injury, whereas those who were not injured landed on the forefoot [11]. Another study showed that sagittal plane foot position at initial contact altered hip and knee joints flexion, and knee joint abduction angles [12]. Bringing the existing evidence together, a high-risk landing strategy for ACL injuries would be a combination of rearfoot or flatfooted landing, low hip and knee joints flexion angles, high knee joint abduction angle, high impact forces and knee joint abduction moments. Consequently, it seems that risk of ACL injuries could be minimized with a proper landing strategy.

Recent studies in running have provided evidence that experience induces changes in kinematics and kinetics of running that may help reduce the risk of injury [13, 14]. However, no study has ever compared landing strategy across different groups of volleyball experience. Therefore, the purpose of the current study was to compare the kinematics and the kinetics of the lower limb between experienced and novice volleyball players during block and spike jumps. It was hypothesized that experienced volleyball players would land with larger plantarflexion, knee and hip flexion angles at initial contact and larger dorsiflexion, knee and hip flexion ranges of motion. It was assumed that increasing lower limb joint angles at initial contact and ranges of motion would result in a reduction of the GRF parameters [15–17]. In the frontal plane, it was hypothesized that experienced volleyball players would land with smaller knee abduction angle and moment.

## Methods

### Participants

A total of 30 males (15 experienced and 15 novice) volleyball players participated in the current study (Table 1). The inclusion criteria for experienced players were to practice volleyball at least twice a week and to have more than three years of competitive experience in a French division. The inclusion criteria for the novice players was to have no history of volleyball practice. Participants from both groups had no history of lower-limb injuries 6 months prior the experiment. All participants read and signed an informed consent prior to participating in any study procedure. All methods were carried out in accordance with the Declaration of Helsinki and all the procedures have been approved by the Aix-Marseille University ethics committee.

**Table 1:** Participant characteristics by experience group

	Experienced group	Novice group	*p-value*
Participants (n)	15	15	–
Age (years)	28.7 (7.1)	27.6 (5.4)	0.692
Height (m)	1.8 (0.8)	1.8 (0.7)	0.118
Weight (kg)	80.5 (8.5)	75.7 (9.1)	0.147
Volleyball training (h/week)	6.5 (2.0)	None	–
Volleyball experience (years)	14.0 (7.1)	None	–
Jump height (cm)	44.8 (8.9)	36.4 (7.1)	0.010*

*p-values as revealed by independent t-tests

### Experimental protocol

**Fig. 1:**
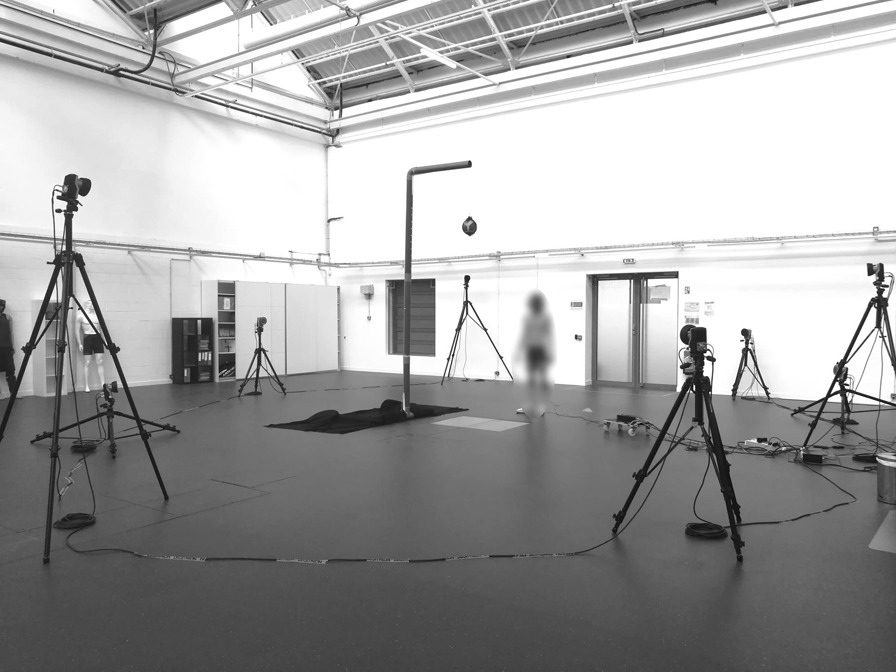
Representation of the experimental environment

First, the participants performed a 5-min self-selected warm-up and a familiarization period with the tasks. After that, they had to complete two maximal effort block jumps. Jump height of each trial was calculated by subtracting the mean height reached by both hands during the jump with the height of the hands in a standing position on tip-toes with arms outstretched towards the ceiling. The maximum jump height among both maximal trials was used to set the target height at 85% of this maximum. Then, participants performed block and spike volleyball jump-landing tasks. For both tasks, participants were asked to complete a three-step approach followed by a two-legged take-off [18]. During the aerial phase, they had to reach a ball with their hand, as they would have done in real practice conditions (Fig. 1). The ball was placed at 85% of the maximum height reached during the two maximal effort block jumps. The block jump was characterized by a simple vertical jump and the smash jump by a vertical and forward jump. During the landing phase, participants were instructed to land on both feet with one foot on a different force plate. The dimensions of the force plates were 60 × 40 cm for the block jump and two 90 × 60 cm for the spike jump. Finally, participants were asked to maintain their balance for 5 s after landing. Eight successful trials of each jump-landing task were performed in random order and with a minimum of 30 s of rest between trials. Participants wore their own personal sport shoes.

### Data collection and processing

**Fig. 2:**
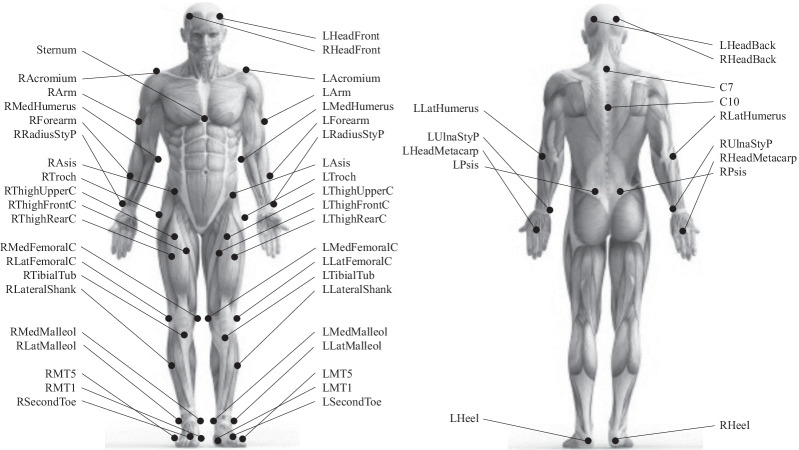
Position of the 55 passive reflective markers used in the study

Participants were instrumented with 55 passive reflective markers with a diameter of 14 mm. Markers were placed on the right and left limbs at the medial and lateral malleolus, lateral shank, medial and lateral femoral condyles, greater trochanter, posterior superior iliac spine, anterior superior iliac spine, third head of metacarpal, radius-styloid process and ulna-styloid process, medial and lateral epicondyle of humerus, arm and acromion. Three markers were placed at the mid-thigh using a rigid cluster. Each shoe was also instrumented with four markers placed on the lateral side of fifth metatarsal head, medial side of first metatarsal head, posterior heel and second toe. Finally, markers were placed on the left and right anterior and posterior head, thoracic vertebrae 10, cervical vertebrae 7 and sternum (Fig. 2).

A motion analysis system composed of 16 cameras (Oqus, Qualisys, Sweden, 200 Hz) and 4 force plates (two 9287CA, one 9281C and one 9281EA, Kistler, Switzerland, 2000 Hz) were synchronized to collect markers trajectories and GRF data, respectively. To avoid possible signal distortion on impact peak, no filtering was carried out on the GRF data. On the other hand, a 10 Hz fourth-order Butterworth low-pass filter with zero time timelag was conducted on the three-dimensional marker coordinates. Then, the joint kinematics and moments through was computed using a full-body musculoskeletal model with 42 degree of freedom and 92 muscles actuators (43 per leg and 6 at the torso) and the OpenSim v4.0 software [19]. The model was scaled to each participant’s dimension, and the lower limb joint angles were calculated from the filtered markers coordinate data using the inverse kinematics tool from OpenSim. Net internal joint moments in the frontal plane were calculated using the inverse dynamic tool from OpenSim combining the kinematic and kinetic data. To determine these moments, equations of motion for the system are solved iteratively. The equations of motion are derived using the kinematic description and mass properties of the musculoskeletal model. Then, using the joint angles from inverse kinematics and ground reaction force data, the net moments at each of the joints are calculated such that the dynamic equilibrium conditions and boundary conditions are satisfied [20]. For this particular step, both kinematic and force plate data were filtered at the same cutoff frequency of 10 Hz using a zero timelag fourth order Butterworth filter to overcome inaccuracies in assessment of joint moments [21]. Kinematic dependent variables were hip, knee and ankle joints flexion angles and knee joint abduction angles at initial contact and range of motion for the landing phase. The landing phase was defined as the time interval between initial foot to ground contact and the maximum knee flexion angle. Initial foot contact was determined when the vertical GRF first exceeded 10 N. Hip, knee joints flexion, ankle joint dorsiflexion and knee joint abduction angles were assigned to be positive. Foot floor angle at initial contact was also compute for a graphical representation. Kinetic dependent variables were composed of the magnitude peak of vertical GRF and its associated loading rate defined by the slope of force–time curve from 20 to 80% before the peak. The GRF variables were normalized to body weight. Peak knee abduction moment for the landing phase was also part of kinetic dependent variables. The knee abduction moment was considered positive and was normalized to the body mass.

### Statistics

First, the normality of residuals using the Shapiro–Wilk test and the homogeneity of variance were checked. Then, a two-sample Student's t-test was used to compare the characteristics of participants by volleyball experience group. A two-way mixed ANOVA with a between-subjects factor of group experience and a within-subjects factor of jump-landing task was also performed on each dependent variable. This analysis was used to investigate the effect of volleyball experience (main effect group) and the interaction between the effect of volleyball experience and the jump-landing task (interaction) on the landing strategy. The alpha threshold was set at *a* = 0.05. Small (0.02 < ωp^2^ < 0.13), medium (0.13 < ωp^2^ < 0.26) and large (ωp^2^ > 0.26) effect sizes were estimated through partial omega squared ωp^2^. RStudio software (version 1.1.453, RStudio, Inc) was used to perform all statistical analyses. A second statistical model using a one-way ANCOVA with jump height as a covariate factor was used to further evaluate the effect of volleyball experience. This second analysis was detailed in the Additional file 1.

## Results

**Fig. 3:**
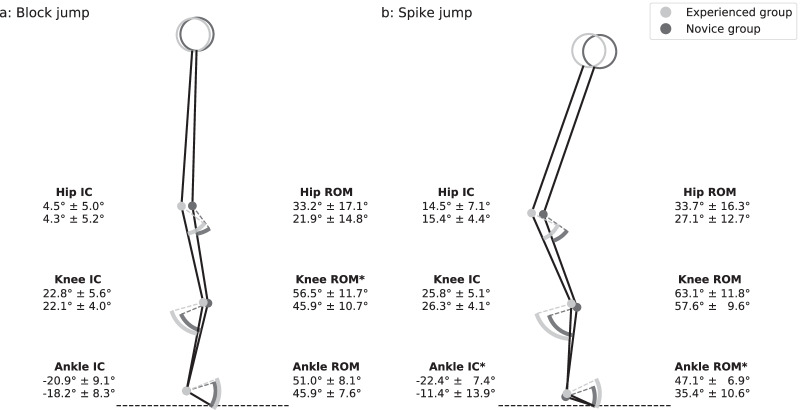
Stick graphic representation of the mean of hip, knee and ankle joints angles at initial contact (IC) in the sagittal plane for experienced and novice volleyball players and for block jump (**A**) and spike jump (**B**). Range of motion (ROM) of hip, knee and ankle joints is represented by pieces of pie chart. Mean ± standard deviation of hip, knee and ankle joints angles at IC and ROM are also reported. ^a^Statistically significant difference between groups (*p*-value < 0.05)

Groups showed no statistically significant difference in age, height and weight (Table 1). Experienced volleyball players jumped statistically significantly higher than novices. The two-way mixed ANOVA revealed a significant main effect of volleyball experience on the ankle dorsiflexion range of motion (*p* = 0.010, ωp^2^ = 0.183, medium effect size). Overall, the experienced group exhibit a larger ankle dorsiflexion range of motion than novices. No statistically significant main effect of volleyball experience on other kinematic and kinetic dependent variables was found (Table 2). The mixed ANOVA revealed significant interaction of volleyball experience and the jump-landing task on ankle plantarflexion angle at initial contact (*p* = 0.001), hip flexion range of motion (*p* = 0.010), knee flexion range of motion (*p* = 0.003) and ankle dorsiflexion range of motion (*p* = 0.003). Specifically, experienced volleyball players landed with significantly more plantarflexion at initial contact than novices for the spike jump (*p* = 0.015, ωp^2^ = 0.160, medium effect size) but no difference was found for the block jump (*p* > 0.05). In addition, the experienced volleyball players had a larger range of motion knee flexion during the block jump (*p* = 0.018, ωp^2^ = 0.150, medium effect size) but not during the spike jump (*p* > 0.05), and a larger range of motion of ankle dorsiflexion during the spike jump (*p* = 0.002, ωp^2^ = 0.268, large effect size) but not during the block jump (*p* > 0.05) compared to novices (Fig. 2). Figure 3 is a boxplot representation of the foot floor angle at initial contact of both jump-landings for experienced and novice volleyball players. This graphical representation provides additional information on the foot strike pattern adopted by each participant in both groups. Finally, statistically significant interactions between the volleyball experience and the jump-landing task were found for the knee abduction angle at initial contact (*p* = 0.036) and the knee abduction angle range of motion (*p* = 0.009) (Table 2). No statistically significant group effect was found on these two variables for block jump and spike jump. However, the jump-landing task influenced the knee abduction angle at initial contact in novices (*p* = 0.002, ωp^2^ = 0.486, large effect size) but not in experienced players (*p* > 0.05). The range of motion of the knee abduction also differed depending on the jump-landing in experienced players (*p* < 0.001, ωp^2^ = 0.774, large effect size) and novices (*p* < 0.001, ωp^2^ = 0.643, large effect size).

**Table 2:** Mean ± standard deviation of kinetic and kinematic parameters for experienced and novice volleyball players and for block and spike jumps

	Experienced group		Novice group		Group effect		Interaction effect	
	Block jump	Spike jump	Block jump	Spike jump	*p-values*	ωp^2^	*p-values*	ωp^2^
Peak vertical GRF^a^ (BW)	2.76 ± 0.74	3.70 ± 1.02	2.83 ± 0.41	3.58 ± 0.64	0.936	< 0.001	0.342	< 0.001
GRF loading rate (BW/s)	67.7 ± 57.9	167.4 ± 88.1	71.9 ± 34.5	162.5 ± 48.9	0.943	< 0.001	0.691	< 0.001
Knee abduction angle at IC^b^ (°)	− 2.1 ± 2.0	− 2.3 ± 1.9	− 3.1 ± 2.8	− 4.0 ± 3.0	0.150	0.038	0.036*	0.113
Knee abduction ROM^c^ (°)	− 5.4 ± 1.8	− 6.6 ± 2.1	− 5.0 ± 1.5	− 7.7 ± 2.6	0.579	< 0.001	0.009*	0.184
Peak knee abduction moment (Nm/kg)	0.21 ± 0.10	0.25 ± 0.13	0.15 ± 0.11	0.16 ± 0.14	0.114	0.053	0.437	< 0.001

^a^Ground Reaction Force

^b^Initial Contact

^c^Range of Motion

*p-values < 0.05

## Discussion

In the present study, experienced and novice volleyball players’ landing kinematics and kinetics during block and spike jump-landings were compared. As hypothesized, volleyball experience influenced the landing strategy used by the participants during both jump-landing tasks. However, the change in kinematics was not observed for all joints of the lower limb and was related to the jump-landing task performed. The only main effect of volleyball experience observed was the increase in ankle dorsiflexion range of motion in the experienced group compared to the novice group. This increase is probably related to a more forefoot approach used by the experienced players during initial foot–ground contact, especially during the landing of the spike jump. A forefoot approach allowed larger dorsiflexion range of motion which likely delayed the heel strike and prolonged the time to the vertical peak force by using the range of motion the ankle joint [22, 23]. In this way, the eccentric work of the ankle plantarflexor muscles may contribute more to the reduction of GRF parameters during a forefoot approach with a large foot floor angle compared to a compared to a smaller angle [24]. Based on our data, it seems that the experienced group used the ankle joint more effectively to better attenuate the vertical GRF compared to the novice group. This result could be used to selectively target the ankle joint in learning exercises for novice volleyball players.

**Fig. 4:**
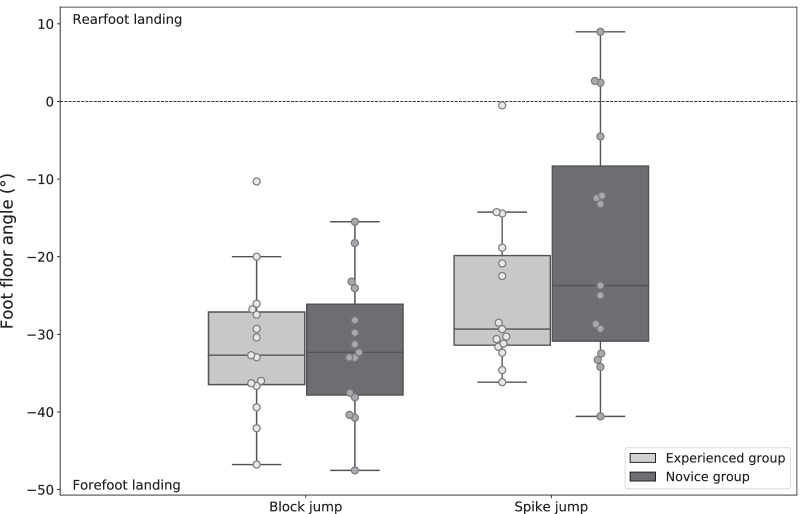
Foot floor angle at initial contact for experienced and novice volleyball players and for block and spike jumps

There are several interactions between the experience of the volleyball players and the jump-landing task performed. First, the experienced volleyball players landed with more plantarflexion at initial contact and larger ankle dorsiflexion range of motion of during the spike jump but not during the block compared to novices. The data distribution indicated that the foot landing pattern used by the experienced group was more consistent than the one used by the novice group (Fig. 4). During the spike jump, most experienced and some novice volleyball players landed on their forefoot with the ankle plantarflexed. However, other novices landed on their midfoot or rearfoot with less ankle plantarflexion. These results suggested that two foot strike patterns were used by novices during spike jump: forefoot and midfoot/rearfoot. Forefoot landing is defined as the first foot–ground contact with the front part of the foot and the rearfoot and midfoot landing correspond to a rear foot and a flat foot contact, respectively. During the block jump, both experienced and novice players landed with a forefoot strategy. Experienced volleyball players appear to use an appropriate foot landing strategy to reduce the risk of ACL injury regardless of the type of jump performed [11]. However, novice players appear to use an appropriate foot landing strategy only during the block jump, and some novices seem to use a high-risk foot landing strategy during the spike jump. Landing with a forefoot strategy after a spike jump may seem counterintuitive to these novice players. The modification of the foot landing strategy may be caused by the forward component found only in the smash jump. Indeed, the aerial phase of the block jump was only on vertical direction and the aerial phase of the spike jump was on both vertical and forward directions. The landing phase of the spike jump can also be considered a breaking phase of the forward component that may facilitate a rear foot or a flat foot contact with the ground in novice players.

In addition, the results revealed that the experienced volleyball players had a larger knee flexion range of motion during the block jump but not during the spike jump. Larger lower limb ranges of motion, particularly at the knee joint, has been reported to decrease the peak vertical peak ground reaction force and prolonged the time to reach the peak value during landing [17, 24]. Moreover, hip and knee joints were previously identified as great contributors to energy absorption during landing [25]. Lower knee range of motion found in the novice group indicate that this joint was not as involved in the impact force attenuation during landing as it was in the experienced group. In the frontal plane, no difference was found in knee abduction angle at initial contact and both groups landed with the knee joints adducted during both block and spike jumps. This observation was consistent with a previous study which reported that university volleyball players landed with their knee joints adducted at initial contact of a block jump [26]. Interestingly, the knee angle in the frontal plane seems to be more affected by the type of jump in novices than in experienced players. Our results also showed no statistically significant difference on peak knee abduction moment for both jumps between groups, potentially revealing that the tasks used were not challenging enough to observe differences in knee abduction angles and moments. A systematic review that investigated the effect of landing height on knee abduction angle as a function of sex reported, for seven out of eight studies, an increase in knee abduction angle or moment on females when performing a drop landing from a height greater than 40 cm whereas two out of three showed no sex differences with a height 30 cm or lower [27]. In the current study, experienced and novice volleyball players landed on average from a height of 44.8 and 36.4 cm, respectively. These results suggested that the jump height of the selected tasks (85% of the maximal jump height) was potentially not sufficient to influence knee abduction angle and peak knee abduction moment in experienced and novice volleyball players.

Landing kinematics may be associated to predisposing factors for an ACL injury. Previous studies highlighted evidence that landed with the midfoot or rearfoot and restricted dorsiflexion range of motion may predispose athletes to ACL injury [11, 23]. Based on these findings, novice volleyball players using midfoot or rearfoot strike pattern seemed to land in a high-risk position that could resulted in an ACL rupture. Additionally, forefoot landing pattern was previously associated with smaller knee flexion angle at initial contact [28]. However, smaller knee flexion angles at initial contact increased GRF in controlled impact conditions and appeared to be a risk factor for ACL injury [10, 15]. In our study, experienced volleyball players landed with larger ankle plantarflexion angles while maintaining knee flexion angles similar to that of novices, which could be the most effective strategy to reduce the potential risk of ACL injury.

Finally, the results revealed that the experienced volleyball players jumped higher and thus landed from a higher height than novices. This observation is consistent with a study that reported higher vertical jump performance in volleyball players with 3 years of experience compared to an inexperienced control group [29]. Interestingly, although experienced volleyball players landed from a higher height compared with novices, no statistically significant difference was found in vertical GRF peaks and loading rates between groups. From a mechanical point of view, an increase in the landing height would lead to an increase in the GRF, however experienced volleyball players seemed to use an efficient load accommodation strategy to reduce this impact force in both jump-landing tasks. Restricted lower limb ranges of motion observed in novice volleyball players may also help to explain the absence of difference in GRF parameters.

The overall findings of the present study support our hypothesis that experienced volleyball players exhibit a different landing strategy compared to novice players. For the block jump, experienced volleyball players used a knee attenuation strategy and for the spike jump, they used an ankle attenuation strategy. These findings suggested that experienced volleyball players adapted their landing strategy to the mechanical demands of each jump-landing tasks. This adaptation seemed to be an effective way to reduce the impact force during landings from higher volleyball jumps. In addition, based on the literature and the present study, experienced volleyball players seem to have the most effective strategy to reduce the potential risk of ACL injury. A possible explanation for the difference in landing technique between experienced and novice volleyball players may be related to the repetition of jumps performed during regular volleyball training and competition. Experienced volleyball players likely intuitively adapted their strategy to reduce the risk of ACL injury, whereas novices did not have enough experience to adopt a low-risk landing strategy. In addition, the volleyball experience appeared to contribute to a more repeatable landing strategy for participants in the experienced group, regardless of the type of jump performed. The practice of a sport involving jumps such as volleyball seemed to induce an adaptation of the landing strategy that would be less risky for ACL injuries compared to athletes with no experience in jumping-related sports.

To reduce injury risk on novices and less experienced volleyball players, special attention should be given to learning a proper landing strategy and the results of the present study can form the basis of the learning exercises. Expert oral and video feedbacks could be an effective way to modify lower extremity kinematics and turn a landing strategy predisposed to an ACL injury into a safer one [30]. The principal limitation of our study was that the jump height of both jump-landing tasks was set at 85% of the maximal jump height of each participant to avoid any fatigue effects, but this height was probably not challenging enough to induce potential differences in knee abduction angles and moments. As this landing height did not induce differences in the knee abduction angle and moment, this value may be used as a training threshold when the players are returning to practice after an ACL injury. Finally, experience may not be the only factor that contributed to differences in landing strategy between experienced and novice volleyball players. Many factors may influence the landing biomechanics such as muscle strength and history of anterior cruciate ligament injury [31, 32].

## Conclusions

In the current study, experienced and novice volleyball players used different landing strategies for both block and spike jump-landings. Overall, the landing strategy seemed to be more affected by the type of jump in novices than in experienced players. Especially, some novices adopted an atypical midfoot/rearfoot strike pattern that likely reduced their ability to attenuate GRF parameters during the spike jump. In this way, our results indicated that lower limb joints were not as involved in the impact force attenuation in the novice group compared to the experienced group during landing in both jumps. Finally, although the experienced group landed from a higher height compared with the novice group, no difference was found in GRF parameters. These findings revealed that the experience of volleyball players acquired during practice of regular trainings and competitions may play an important role in the landing strategy and the attenuation of the impact force, with different strategies depending on the type of jump.

## Supplementary Information

**Additional file 1: Table S1.** Mean ± standard deviation of the dependent variables of interest for experienced and novice volleyball players and for block and spike jumps.

## Data Availability

The datasets generated and analysed during the current study are available in the figshare repository 10.6084/m9.figshare.19728139.

## Abbreviations

ACL: Anterior cruciate ligament.
GRF: Ground reaction force
