# Supplementary material for: Comparison of landing kinematics and kinetics between experienced and novice volleyball players during block and spike jumps
Source: BMC Sports Sci Med Rehabil. 2022 Jun 11;14:105. doi: 10.1186/s13102-022-00496-0 (PMC9188216; doi:10.1186/s13102-022-00496-0)
Supplement: Supplementary file 1 — Additional file 1: Table S1. Mean ± standard deviation of the dependent variables of interest for experienced and novice volleyball players and for block and spike jumps. [file 13102_2022_496_MOESM1_ESM.docx]

**Background**

While the jump height of each participant was experimentally controlled relatively to their individual jump ability, the absolute jump height differences between participants can have potential effects on kinematic and kinetic variables (Ali, Robertson and Rouhi, 2014; Peng et al., 2019).

**Statistics**

The expertise effect was evaluated through a second statistical model using a one-way ANCOVA with jump height as covariate factor. A permutation test was performed when the assumptions for ANCOVA were not satisfied. The significance level was set at p<0.05. Cohen's d was used to determine small (0.2-0.5), medium (0.5-0.8) and large (>0.8) effect sizes. All statistical analyses were performed using RStudio software (version 1.1.453, RStudio, Inc).

**Results**

For the block jump, the ANCOVA revealed no statistically significant difference was between groups on each kinematic and kinetic variable (Table 1). For the spike jump, the ANCOVA showed statistically significant differences with a large effect size on ankle dorsiflexion angle at initial ground contact (*p* = 0.030, *d* = 0.988) and on ankle dorsiflexion range of motion (*p* = 0.018, *d* = 1.308), and a medium effect size on knee flexion range of motion (*p* = 0.038, *d* = 0.511) and on knee adduction angle at initial ground contact (*p* = 0.040, *d* = 0.677). Experienced volleyball players landed with statistically significant larger ankle plantarflexion angle at initial contact, ankle dorsiflexion and knee flexion range of motions, and statistically significant lower knee adduction angle at initial contact compared to novices. No statistically significant difference was found between groups on the other kinematic of kinetic variables.

|  | Bock jump | | | Spike jump | | |
| --- | --- | --- | --- | --- | --- | --- |
|  | Experienced  group | Novice  group | ANCOVA  *p-values* | Experienced  group | Novice  group | ANCOVA  *p-values* |
| Hip flexion angle IC^a^ (°) | 4.5 ± 5.0 | 4.3 ± 5.2 | 0.736 | 14.5 ± 7.1 | 15.4 ± 4.4 | 0.222 |
| Knee flexion angle IC (°) | 22.8 ± 5.6 | 22.1 ± 4.0 | 0.951 | 25.8 ± 5.1 | 26.3 ± 4.1 | 0.397 |
| Ankle dorsiflexion angle IC (°) | -20.9 ± 9.1 | -18.2 ± 8.3 | 0.510 | -22.4 ± 7.4 | -11.4 ± 13.9 | 0.030* |
| Hip flexion ROM^b^ (°) | 33.2 ± 17.1 | 21.9 ± 14.8 | 0.765 | 33.7 ± 16.3 | 27.1 ± 12.7 | 0.052 |
| Knee flexion ROM (°) | 56.5 ± 11.7 | 45.9 ± 10.7 | 0.735 | 63.1 ± 11.8 | 57.6 ± 9.6 | 0.038* |
| Ankle dorsiflexion ROM (°) | 51.0 ± 8.1 | 45.9 ± 7.6 | 0.681 | 47.1 ± 6.9 | 35.4 ± 10.6 | 0.018* |
| Knee abduction angle at IC (°) | -2.1 ± 2.0 | -3.1 ± 2.8 | 0.217 | -2.3 ± 1.9 | -4.0 ± 3.0 | 0.040* |
| Knee abduction ROM (°) | -5.4 ± 1.8 | -5.0 ± 1.5 | 0.775 | -6.6 ± 2.1 | -7.7 ± 2.6 | 0.689 |
| Peak vertical GRF^c^ (BW) | 2.76 ± 0.74 | 2.83 ± 0.41 | 0.203 | 3.70 ± 1.02 | 3.58 ± 0.64 | 0.673 |
| GRF loading rate (BW/s) | 67.7 ± 57.9 | 71.9 ± 34.5 | 0.307 | 167.4 ± 88.1 | 162.5 ± 48.9 | 0.179 |
| Peak knee abduction moment (Nm/kg) | 0.21 ± 0.10 | 0.15 ± 0.11 | 0.598 | 0.25 ± 0.13 | 0.16 ± 0.14 | 0.728 |

Table 1. Mean ± standard deviation of the dependent variables of interest for experienced and novice volleyball players and for block and spike jumps.

a Initial Contact. b Range of Motion. c Ground Reaction Force. * p-values < 0.05.
